# Tiny sensors, big hope: ML-optimized nanodiagnostics for TBI in Sanfilippo syndrome

**DOI:** 10.1097/MS9.0000000000003781

**Published:** 2025-08-28

**Authors:** Maria Feroz, Shifa Ahmad, Maliha Khalid, Muhammad Talha, Aminath Waafira

**Affiliations:** a Khyber Girls Medical College, Peshawar, Pakistan; b Jinnah Sindh Medical University, Karachi, Pakistan; c King Edward Medical University, Lahore, Pakistan; d School of Medicine, The Maldives National University, Malé, Maldives

**Keywords:** machine learning, nanodiagnostics, sanfilippo syndrome, traumatic brain injury

## Abstract

Sanfilippo syndrome (mucopolysaccharidosis type III) is a rare autosomal recessive disorder marked by neurodegeneration due to defective heparan sulfate metabolism. Diagnostic and therapeutic limitations persist, particularly for detecting comorbid conditions like traumatic brain injury (TBI), which requires sensitive, rapid tools for timely intervention. Emerging ML-optimized nanodiagnostic platforms offer a breakthrough, allowing early-stage, high-fidelity detection of TBI biomarkers in biofluids. Recent studies employing surface-enhanced Raman spectroscopy (SERS) and multiplex electrochemiluminescence (ECL) sensors demonstrate the capacity for real-time, low-volume, point-of-care TBI diagnosis. However, limitations remain in adapting these models for rare pediatric populations due to data scarcity, blood-brain barrier constraints, and biomarker kinetics. We call for expanded validation, interdisciplinary collaboration, and the development of wearable diagnostics to personalize care and improve outcomes in Sanfilippo syndrome and beyond.

*To the Editor,*

Sanfilippo syndrome (mucopolysaccharidosis type III) is an autosomal recessive disorder caused by abnormalities in an enzyme involved in the metabolism of heparin sulfate. The central nervous system is primarily targeted by the ensuing accumulation of mucopolysaccharides, resulting in impairment of speech, cognitive, motor, and behavioral functions. Symptoms usually manifest in early childhood and worsen at an accelerated pace, progressing to severe neurological deficit and early-onset mortality. Diagnosis relies on clinical and biochemical testing, and there is currently no therapeutically beneficial approach despite advancements in understanding its biochemical and genetic determinants. The detection of secondary insults like traumatic brain injury is even more challenging in this scenario, requiring sensitive diagnostic methods^[1]^.

Currently, the emerging approach towards Sanfilippo syndrome is primarily focused on enzyme replacement and gene therapy, restoring the deficient enzyme and attenuating the neurodegeneration. However, these therapies have some limitations, such as ineffective penetration across the blood-brain barrier, requiring novel CNS targeting strategies. Diagnostic modalities incorporating advanced brain imaging techniques, including MRI and EEG remains crucial, yet the high sensitivity of Machine learning (ML)-optimized nanodiagnostics in detecting the subclinical pathological alterations in bio-fluids and refining disease stratification, permit prompt interventions for conditions like traumatic brain injury in Sanfilippo syndrome, where timely intervention is essential before irreversible degeneration can occur. Such diagnostic approaches are promising beyond Sanfilippo syndrome, with application spanning to other neurodegenerative disorders such as multiple sclerosis and Alzheimer’s disease^[2]^.

Recent clinical researches showcase the diagnostic efficacy and accuracy of Nanodevice-based diagnostic systems in the early detection of traumatic brain injury (TBI). The 2024 study by Gao et al. developed a gold nanorod–based SERS biosensor, which, by ultra-sensitive detection of TBI biomarkers S100β and IL-6 in animal and human serum, offers precise quantification of biomarkers^[3]^. In parallel to this, an accurate, low-cost, point-of-care diagnosis of mild TBI from small blood samples is enabled through a portable multiplex ECL-based biosensor using screen-printed carbon electrodes, which was designed to detect GFAP, S100β, and h-FABP (major TBI biomarkers) under 15 min^[4]^. Collectively, these studies portray the clinical potential of nanosensors in transforming the assessment of early-stage TBI through rapid, sensitive, and reliable means.

While these innovations are promising, several limitations persist. The rarity and phenotypic heterogeneity of Sanfilippo syndrome limit the generalizability of ML-optimized nanosensor models because most models are trained on data from adults, which means they don’t work as well for children or rare cases^[5]^. Key obstacles that limit the integration of real-time biosensing for brain injury diagnosis are the blood-brain barrier, a potential technical barrier that may hinder many central nervous system biomarkers from crossing it, and rapid degradation or clearance of these biomarkers in circulation, complicating reliable detection at clinically meaningful timepoints^[6]^. Our work is in line with the TITAN Guidelines on the need for transparency in AI use in healthcare^[7]^.

In conclusion, the ML-optimized nanodiagnostics hold a significant avenue, and by exploring the link between rare neurodegenerative disorders and the trauma-induced pathophysiology, this advancement can yield significant improvement. Advancement of this paradigm calls for future efforts that should focus on large-scale validation in a broader population, supporting multidisciplinary collaborations and, most importantly, integrating wearable diagnostic systems, which would allow more personalization and real-time decision making in detecting an early TBI.

## Data Availability

No data were generated for this manuscript.

## Peer and provenance statement

This article was not commissioned and was externally peer-reviewed.
